# Wavefunction engineering towards high-performance terahertz quantum cascade lasers

**DOI:** 10.1038/s41598-025-10080-4

**Published:** 2025-08-11

**Authors:** Seyed Ghasem Razavipour

**Affiliations:** Quantum and Nanotechnologies Research Center, National Research Council Canada, 1200 Montreal Road, Ottawa, ON K1A 0R6 Canada

**Keywords:** Quantum cascade lasers, Terahertz optics

## Abstract

In the quest for high-performance terahertz (THz) quantum cascade lasers (QCLs), this study introduces a generalized wavefunction engineering approach to efficiently control state populations at elevated temperatures. Analyzing known two-well structures and their limitations, a three-well QCL design based on a direct depopulation scheme is proposed. Employing a combination of rate equations-density matrix and NEGF modelings, our design achieves superior performance at 290 K by simultaneously optimizing injection coupling, thermal back-filling, and electron escape rates from upper and lower lasing states to parasitic states.

## Introduction

The Terahertz (THz) electromagnetic spectrum (1 - 10 THz) has garnered significant interest in research circles due to its diverse applications including but not limited to biomedicine^1,2^, security control^3,4^, high-speed short-distance communication^5^, astronomy^6,7^, and spectroscopy^8,9^. The primary barrier hindering the advancement of commercial imaging and spectroscopy systems in the terahertz field is the absence of high-performance, room-temperature THz sources. This limitation significantly slows progress by restricting the practical deployment and scalability of these technologies in various applications. THz quantum cascade lasers (QCLs), first demonstrated in 2002^10^, have achieved significant breakthroughs over the past two decades, including substantial enhancements in maximum output power at low temperatures in both pulsed^11^ and continuous-wave modes^12^, expansion of their operational frequency range from 1.2 THz^13^ to 5.7 THz^14^, and refined beam pattern engineering^15^. Despite these advancements, the progress in the operating temperatures of these lasers has not kept pace with other developments. Although the improvement in the operating temperatures of THz QCLs to 250 K^16^ and 261 K^17^, eliminates the need for cryogenic cooling, these developments still fall short of the minimum specifications required for commercial applications, such as milliwatt power output at room temperature. This gap highlights the ongoing challenges in meeting the practical demands of the industry. Recently published high-performance QCL designs underscore the importance of compact, comprehensive, and accurate theoretical modeling. This modeling is an essential prerequisite for the effective practical implementation, optimization of existing structures, and deeper understanding of the underlying physical processes involved. The Wigner function formalism or the non-equilibrium Green’s function (NEGF) model, which includes the most comprehensive treatment of scattering from all relevant processes, is capable of accurately simulating experimental devices^18–20^. However, the computationally expensive process of gain analysis using the NEGF simulator makes this model not the best option for the optimization of quantum structures. The rate equation (RE) transport model, which is commonly used in the design and analysis of mid-infrared QCL, benefits from the fast calculation process with a cost of non-physical results in some THz QCL structures due to the lack of including the coherent quantum mechanical transport effect^21^. The density-matrix (DM) model as a powerful transport model has been improved by including the infinite-period approach and well-defined eigenstates^22^ instead of manually-chosen tight-binding states^23^ with some computational and numerical costs. Self-consistent density matrix ensemble Monte Carlo (DM-EMC) modeling has also been demonstrated as a powerful and accurate tool for estimating charge carrier transport in QCL, effectively capturing both quantum-coherent tunneling and incoherent scattering processes essential to QCL operation^24,25^. In this work, we use the extended rate equation model presented in ref^26^ by including the tunneling rate from the DM formalism called rate equation-density matrix (RE-DM) modeling. A further improvement has been added to the model by calculating the empirical pure dephasing time between the resonant state and the estimation of optical linewidth calculated according to the intrasubband scattering rate based on Ando’s theory^27^. The results proposed by the current model show a good agreement with experimental data and also the simulation results from the commercial software e.g. NextNano.NEGF^28^.

**Figure 1:**
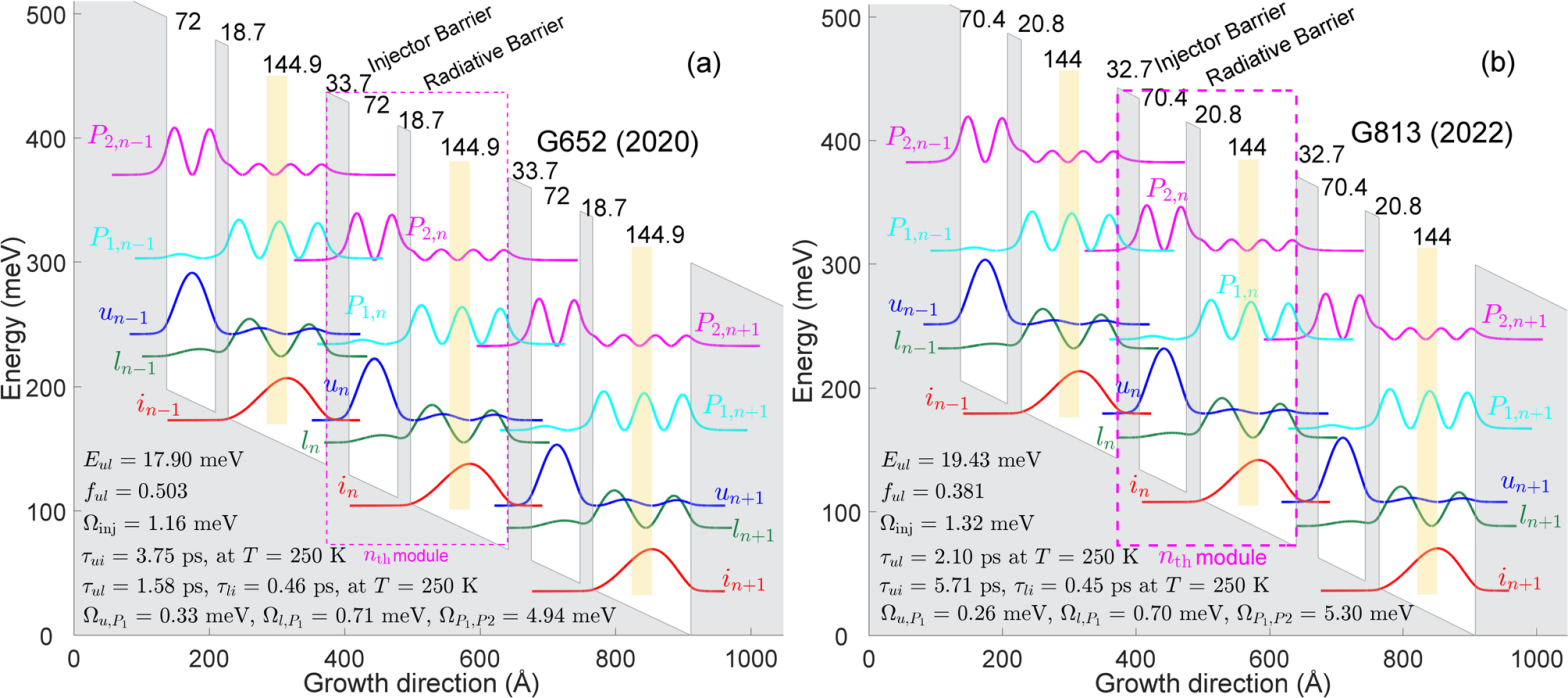
Conduction band diagram and moduli-squared wavefunctions of three neighboring modules at alignment electric fields for the THz QCL active region based on the direct phonon scheme, (**a**) G652^16^ and (**b**) G813^17^. The thickness of each quantum well and barrier, in Angstr$\mathrm {\ddot{o}}$m, is indicated above the graph. The central 3 nm of the largest well is n-doped with 4.5 $\times$ $\mathrm {10^{10}\ cm^{-2}}$ (yellow section).

Proposing a new design scheme or optimization of quantum structure in a well-known design like three-well resonant-phonon^29^, scattering-assisted^30–32^ or direct-phonon (DP) depopulation^33^ schemes can be considered as different methods of wavefunction engineering in a hope to maximize the gain of the structure. This can be achieved by different mechanisms including minimizing the thermal back-filling^34^, controlling the escaping rate of the electrons from the upper lasing state to excited states or the continuum bands^35^, minimizing the linewidth^36^, efficient electron injection to the upper lasing state and selective extraction of the electron from the lower lasing state^37^. Since 2012, when the three-well resonant-phonon design scheme reached its maximum operating temperature at $\sim$200 K^37,38^, almost all high-performance designs ($T_\textrm{max}$ > 200 K) utilized the direct-phonon depopulation scheme based on a two-well quantum structure; examples of which are illustrated in Fig. 1. There are a few characteristics in the two-well QCL structure that attract QCL designers to investigate it in detail and engineer the design parameters. The simultaneous alignment of injection and extraction states at the same electric field in resonant-phonon designs can be disrupted by band-bending effects in doped regions, which are induced by the Poisson effect^39^. However, the direct population scheme involves alignment between only two levels—*i* (the injector state) and *u* (the upper lasing state) as shown in Fig. 1—and therefore, the effect of band bending on laser performance is minimal. The DP design scheme has the lowest possible number of quantum states per period (three states) to achieve the laser dynamics. Moreover, the effect of interface roughness scattering on the performance of the laser may not be significant due to having only four interfaces. References^16,17,40^ analyzed the key features of high-performance designs based on a two-well structure and proposed some approaches to improve the gain and, consequently, the operating temperature of the laser. In Ref^16^, the escaping rate of the electron to the continuum band and the thermal back-filling rate are significantly reduced by employing a higher barrier (high Aluminum concentration) and larger phonon energy spacing ($\sim$50 meV vs. $\sim$36 meV), respectively. Later, a lower oscillator strength and a higher injection coupling were used to improve the operating temperature even further^17^. The same strategy was proposed and theoretically analyzed using an in-house NEGF simulator^41^. In all proposed designs in Refs.^16,17,41^, the levels $P_{\textrm{1}}$ (first parasitic state) and $P_{\textrm{2}}$ (second parasitic state) are highly coupled ($\mathrm {\hbar \Omega }$ = $\sim$5-6 meV, anticrossing energy = $\sim$10-12 meV), and both are partially aligned with upper and lower lasing states of the previous module. Utilizing the phonon energy spacing of $\sim$50 meV and the lasing frequency of 4 THz (A lasing frequency near 4 THz has been historically chosen to achieve the highest operating temperatures, as it offers an optimal trade-off between reduced waveguide losses and the shorter upper-state lifetime associated with higher photon energies^42^) left designers a small room for wavefunction engineering and controlling the levels $P_{\textrm{2}}$ and $P_{\textrm{1}}$ which are the second and the third states of the radiative well and the phonon well, respectively.

In this study, the main drawbacks of the two-well design will be addressed and analyzed using our in-house RE-DM formalism. A wavefunction engineering approach is employed to identify a three-well structure based on a direct phonon depopulation scheme, and its performance will be compared with the best two-well structures proposed so far. The analysis of the proposed structure using NextNano.NEGF shows results comparable to our RE-DM formalism, suggesting that room temperature operation can be achieved in the modified DP structure. This demonstrates that our in-house RE-DM formalism is a robust and reliable tool for optimizing and exploring all potential quantum structures, owing to its parallel and rapid implementation. In contrast, the NEGF method, while powerful, is not suitable for this purpose because of its computationally intensive process.

## Results

The RE-DM formalism employed in this study is the modified version of the model utilized in Ref.^26^ which is well described in^43^. There are three main modifications in our RE-DM model that can affect the accuracy of the calculated current density, the estimated optical gain, and the calculation time using in-house MATLAB code. Coherent transport, typically addressed using the density matrix method and NEGF, is incorporated into the rate equation by introducing the tunneling time model, as shown in Equation 1.

1 $$\begin{aligned} T_{\textrm{Tun}} = (1 + \Delta ^2 \tau _{\parallel }^2)/ 2\Omega ^2 \tau _{\parallel } \end{aligned}$$

where $\mathrm {\Delta }$ and $\mathrm {\Omega }$ are the detuning and coupling energy between two states, calculated using the method described in Ref.^44^, and $\mathrm {\tau _{\parallel }}$ is the dephasing time between the two states. The accurate calculation of the coupling, intersubband scattering time, and pure dephasing time is necessary to estimate the rate of electron injection and, consequently, the population of different states. The detailed calculation method for coupling strength is presented in Ref^43^, the accuracy of which will be discussed in the supplementary material. In addition, the constant pure dephasing time between all aligned states in two neighboring modules in the previous model is replaced by a variable (Voltage and temperature dependent) parameter calculated using the method presented in^27,45^. The second modification is the inclusion of dephasing time between the lasing states as an estimation for the gain bandwidth of the quantum structure. The final modification that has the main impact on this study and facilitates the search of all possible structures without using any optimization process is the parallel implementation of all the functions and subroutines in the MATLAB code. This allowed us to simultaneously simulate different quantum designs (in our case, 500 structures in each run, which is limited by the physical memory) and reduce the calculation time per quantum design. The average run time of one structure, including the calculation of wavefunctions, intra-module scattering time, inter-module tunneling time, current density, and gain spectrum, is less than one second on a personal computer with Xeon(R) CPU E5-2660 at 2 GHz ($\sim$420 seconds for 500 structures).

### State-of-the-art two-well DP structures

In this section, two THz QCLs based on the DP scheme (G652^16^ and G813^17^) are presented and the effects of different design parameters on the performance of the structures are investigated. Figure 1 shows a conduction band diagram and moduli-squared wavefunctions of three neighboring modules. Electrons in the injector level ($i_{n-{\textrm{1}}}$), which contains the majority of the carriers, will pass through the injector barrier via a resonant tunneling process and populate the upper lasing state ($u_n$). A relatively thick radiative barrier is chosen to increase the population inversion between the upper ($u_n$) and lower ($l_n$) lasing states at high temperatures. The depopulation of the level $l_n$ can be achieved using a fast longitudinal optical (LO) phonon process. One of the main advantages of the presented structures compared to the previous two-well designs^33,46^ is the relatively large energy spacing ($\sim$50 meV) between the lower lasing state and the injector state which is far from the LO-phonon energy of GaAs ($\sim$36.7 meV). It improves the population inversion by two different mechanisms, 1-reducing the back-filling rate from level $i_n$ to level $l_n$ and 2- increasing the scattering time between levels $u_n$ and $i_n$. Even though the main objective of this design scheme is to propose a clean three-level structure, the effect of the parasitic states ($P_{{\textrm{1}},n}, P_{{\textrm{2}},n{\mathrm {+1}}}$) cannot be ignored due to their wavefunction overlap with the lower lasing and upper lasing states ($l_{n{\mathrm {-1}}}, u_{n-{\textrm{1}}}$) of the previous module. At aligned electric field (the electric field at which levels $i_{n-1}$ and $u_n$ are in resonance), levels $P_{1,n}$ and $P_{2,n+1}$ are approximately aligned (detuning energy between the two states is less than their coupling energy, $\Delta _{P_{1,n},P_{2,n+1}} < \Omega _{P_{1,n},P_{2,n+1}}$), highly coupled ($\hbar \Omega _{P_{1,n},P_{2,n+1}}\ \approx$5 meV), and generating a miniband with anti-crossing separation energy of $\sim$10 meV ($2\hbar \Omega$). The existence of these parasitic states and their locations limit the optimization process of two-well structures. On the one hand a thinner injector barrier guarantees the coherent injection at high temperature, on the other hand it increases the tunneling rate from states $u_{n-1}$ and $l_{n-1}$ to the parasitic states ($P_{1,n},P_{2,n+1}$). The figure of merit defined in Ref^17^ ($\zeta = \frac{\Omega _{i_{n-1},u_n}}{\Omega _{u_n,P_{1,n+1}}}$) is an attempt to improve the performance of the laser by simultaneously controlling the injection efficiency to the upper lasing state and reducing the leakage to the parasitic state by thinning the injector barrier and selecting a thicker radiative barrier, respectively. The evidence of this implementation can be quantitatively observed by comparing the correct injection ($\Omega _{iu}$ = 1.32 meV in G813 vs. $\Omega _{iu}$ = 1.16 meV in G652), the wrong injection ($\Omega _{il}$ = 0.28 meV in G813 vs. $\Omega _{il}$ = 0.32 meV in G652), the oscillator strengths (*f* = 0.5 in G652 Vs. *f* = 0.38 in G813), and the coupling between the upper lasing state and the parasitic state ($\Omega _{u,P_1}$ = 0.26 meV in G813 vs. $\Omega _{u,P_1}$ = 0.33 meV in G652) in designs G652 and G813. It is noteworthy to mention that the oscillator strength calculated in this study is based on the method in Ref.^47^ and explained in the Methods. The relatively low improvement in temperature performance of the laser ($T_\textrm{max} = \mathrm {260\ K}$ in G813 vs. $T_\textrm{max} = \mathrm {250\ K}$ in G652) comes from fundamental limitation of two-well design in which the position of $P_1$ (the third state of the phonon well) and $P_2$ (the second state of the radiative well) are almost locked and imposed by small possible change in the thickness of the phonon and radiative wells, respectively. All high-performance two-well structures proposed so far suffer from the same problem (Supplementary material). Historically, the lasing frequency of $\sim$4 THz has been used for the design and implementation of high performance (high operating temperature) THz QCLs. Any attempt to push level $P_1$ to a higher energy (narrowing the phonon well) will increase the energy spacing between level *l* and *i*. Thereby, the radiative well has to be thinner to keep the lasing frequency close to $\sim$4 THz. Finally, an increase in the design electric field, which is unavoidable to align the levels $i_{n-1}$ and $u_{n}$, will keep level $P_{1,n+1}$ relatively the same position compared to levels $u_n$ and $l_n$.

**Table 1:** Comparison of two-well DP designs (G552^16^, G652^16^, and VA1030^17^) fabricated at Massachusetts Institute of Technology (MIT) in terms of the thickness of the phonon well ($P_\textrm{w}$), phonon energy spacing ($E_{li}$), thickness of the radiative well ($R_\textrm{w}$), design electric field ($E_\textrm{F}$), detuning energy between the upper lasing state (ULS) and $P_1$ ($\Delta _{u_{n-1},P_{1,n}}$), coupling strength between ULS and $P_1$ ($\Omega _{u_{n-1},P_{1,n}}$), detuning energy between the $P_1$ and $P_2$ ($\Delta _{P_{1,n},P_{2,n+1}}$), coupling strength between $P_1$ and $P_2$ ($\Omega _{P_{1,n},P_{2,n+1}}$).

	$P_\textrm{W}$	$E_{li}$	$R_\textrm{W}$	$E_\textrm{F}$	$\Delta _{u_{n-1},P_{1,n}}$	$\Omega _{u_{n-1},P_{1,n}}$	$\Delta _{P_{1,n},P_{2,n+1}}$	$\Omega _{P_{1,n},P_{2,n+1}}$
G552^16^	15.5 nm	46 meV	7.67 nm	22.5 kV/cm	9.3 meV	0.28 meV	0.4 meV	4.2 meV
G652^16^	14.5 nm	51 meV	7.2 nm	25.6 kV/cm	8 meV	0.33 meV	1.5 meV	4.94 meV
VA1030^17^	13.7 nm	55.7 meV	6.8 nm	28.8 kV/cm	8 meV	0.37 meV	3.3 meV	5.8 meV

An example of the experimental implementation of this attempt is the change in the thickness of the phonon well from 15.5 nm in G552^16^ to 14.5 nm in G652^16^ and ultimately 13.7 nm in VA1030^17^. Table 1 shows the details of the changes in phonon well ($P_\textrm{W}$), the energy spacing between levels $l_n$ and $i_n$ ($E_{li}$), radiative well ($R_\textrm{W}$), the alignment electric field ($E_\textrm{F}$), the detuning ($\Delta _{u_{n-1},P_{1,n}}$) and the coupling energies ($\Omega _{u_{n-1},P_{1,n}}$) between the upper lasing state ($u_{n-1}$) and the first parasitic state ($P_{1,n}$), and the detuning ($\Delta _{P_{1,n},P_{2,n+1}}$) and the coupling ($\Omega _{P_{1,n},P_{2,n+1}}$) energies between the first parasitic state ($P_{1,n}$) and the second parasitic state ($P_{2,n+1}$). A change of approximately 5% in the thickness of each module led to a $\sim$7.5% increase in the energy drop per module of the cascade laser and required a $\sim$12% increase in the design electric field to maintain the alignment between levels $i_{n-1}$ and $u_{n}$. Nevertheless, the detuning energy between levels $u_n$ and $P_{1,n+1}$ remained almost constant (8-9 meV), demonstrating that there is a minimal opportunity for wavefunction engineering within the two-well direct phonon scheme.

Another parameter that can affect the performance of the two-well DP structures is the leakage path from level $u_n$ to level $i_n$. The leakage path from level $u_n$ to level $i_n$ reduces the effective lifetime of the laser and, consequently, the population inversion. At a temperature close to $T_\textrm{max}$ where the effect of stimulated emission can be ignored, a simplified estimation of this leakage can be defined with an expression in equation 2 assuming the total intra-module scattering time of level $u_n$ is mostly determined by the escaping rate of electron to levels $l_n$ and $i_n$ (The energy spacing between level $u_n$ and $P_{1,n}/P_{2,n}$ is high).

2 $$\begin{aligned} I_{\textrm{leak}} \varpropto \tau _{ui}^{-1}/ (\tau _{ui}^{-1}+\tau _{ul}^{-1} ) \end{aligned}$$

The scattering time between the levels $u_n$ and $i_n$ depends on the energy spacing between those states and their wavefunction overlaps. Employing a thinner phonon and radiative wells in G813 increased the energy spacing by approximately 2 meV compared to G652 which may not be very effective in reducing the leakage current. However, increasing the radiative barrier by 10% (2.08 nm in G813 vs. 1.87 nm in G652) can play a role in wavefunction overlap and reduce the leakage current from level $u_n$ to $i_n$. The calculated numbers from equation 2 show that 24.2% of electrons from level $u_n$ will be relaxed to level $i_n$ in G813, whilst this leakage is 27% in G652. Table S.2 in the supplementary material presents the calculated leakage at a temperature close to $T_\textrm{max}$ for several high-performance two-well DP structures published since 2019. By fixing the lasing frequency at $\sim$4 THz and employing an approximately 50 meV phonon spacing (to reduce the back-filling), the only design parameter that controls this leakage current will be the radiative barrier. However, there is a trade-off between lowering the leakage current and maintaining a minimum oscillator strength to satisfy the high enough gain required for lasing at high temperatures.

### Three-well DP structures

The aforementioned discussion on two-well DP structures and a comparison between two high-performance lasers based on $\mathrm {GaAs/Al_{0.3}G_{0.7}As}$ material system suggest that a more complex design with the same number of state per module will give QCL designers the freedom to engineer the wavefunction and control different leakage currents in a hope to increase the gain of the lasers and improve the operating temperature. In this study, we propose a double-well phonon design as an alternative to the single-well phonon used in high-performance DP structures to increase the design complexity and open a road map for the wavefunction engineering approach. Double-well phonon scheme has been used in resonant-phonon structures^48–50^, direct-phonon design^51,52^, and more intensively in scattering-assisted designs^31,53–55^. Contrary to single-well phonon in which energy spacing between level $P_1$ and level *l* cannot be controlled independent of the phonon energy ($E_{li}$), the double-well phonon gives us a variety of options to engineer the position of $P_1$ for a fixed phonon energy. Table 2 presents the design parameters of four DP structures using double-well phonon with approximately the same phonon ($\sim$50 meV) and photon ($\sim$18 meV) energies based on $\mathrm {GaAs/Al_{0.3}Ga_{0.7}As}$ material system.

**Table 2:** Four THz QCL structures based on the three-well DP design scheme, along with their key features, including the phonon and photon energies, the detuning energy between ULS and $\mathrm {P_1}$, and energy gap between the lower lasing state (LLS) and state $\mathrm {P_1}$.

	Quantum structure (Å)	$E_\textrm{F}$(kV/cm)	$E_{li}$(meV)	$E_{ul}$(meV)	$\Delta _{u_{n-1},P_{1,n}}$(meV)	$E_{P_1l}$ (meV)
Example 1	**39.3**/56.8/**24.8**/57.4/**5.2**/62	27.8	50.1	18.1	-23	109
Example 2	**30.5**/61.5/**23.8**/100/**5.4**/39	26.4	50.5	18.2	18.9	68
Example 3	**39.4**/58.6/**25**/59.2/**7.2**/74	26.6	50.3	18.1	-3.4	89.9
Example 4	**34**/68.6/**22**/53/**5**/100	24.2	50.4	18	28	58.3

The energy spacing between the first, second and third energy states in the double-well phonon and their wavefunctions can be engineered by changing the thickness and the position of the middle barrier between the two quantum wells. Even though all four structures have the same phonon transition energy (the energy difference between the second state and the first state in the double-well phonon is $\sim$50 meV), they benefit from a totally different energy separation between the third ($P_1$) and second (*l*) energy states in the double-well phonon. The energy spacings between the third and the second energy states are 109, 68, 89.9, and 58.3 meV in designs Example1, Example2, Example3, and Example4, respectively. However, its equivalent energy separation in structure G652 is 78.8 meV which is imposed by the thickness of the single-well phonon. Comparing the detuning energy between the upper lasing state of the previous module ($u_{n-1}$) and the third energy of the double-well phonon ($P_{1,n}$) in Table 2 shows that the position of $P_1$ can be far above ($\Delta _{u_{n-1},P_{1,n}}$ = -23 meV), far below ($\Delta _{u_{n-1},P_{1,n}}$ = 18.9 meV), almost align with ($\Delta _{u_{n-1},P_{1,n}}$ = -3.4 meV) the upper lasing state, or even far below the LLS of previous module ($\Delta _{u_{n-1},P_{1,n}}$ = 28 meV). Obviously, Example1 with its largely negative $\Delta _{u_{n-1},P_{1,n}}$ will behave as a clean 3-state laser structure, however its gain at high temperature is much lower than Example2 and 4, which is explained in the supplementary material. It is noteworthy to mention that in all high-performance two-well DP structures demonstrated so far, the third state in the phonon well ($P_1$) always falls between the upper and lower lasing of the previous module ($\Delta _{u_{n-1},P_{1,n}}$ = 8-9 meV as presented in Table 1). The effect of employing a double-well phonon in structures presented in Table 2 can be visually observed in Figure S.2 of the supplementary material in which the details of the design parameters and band diagram are illustrated.

**Figure 2:**
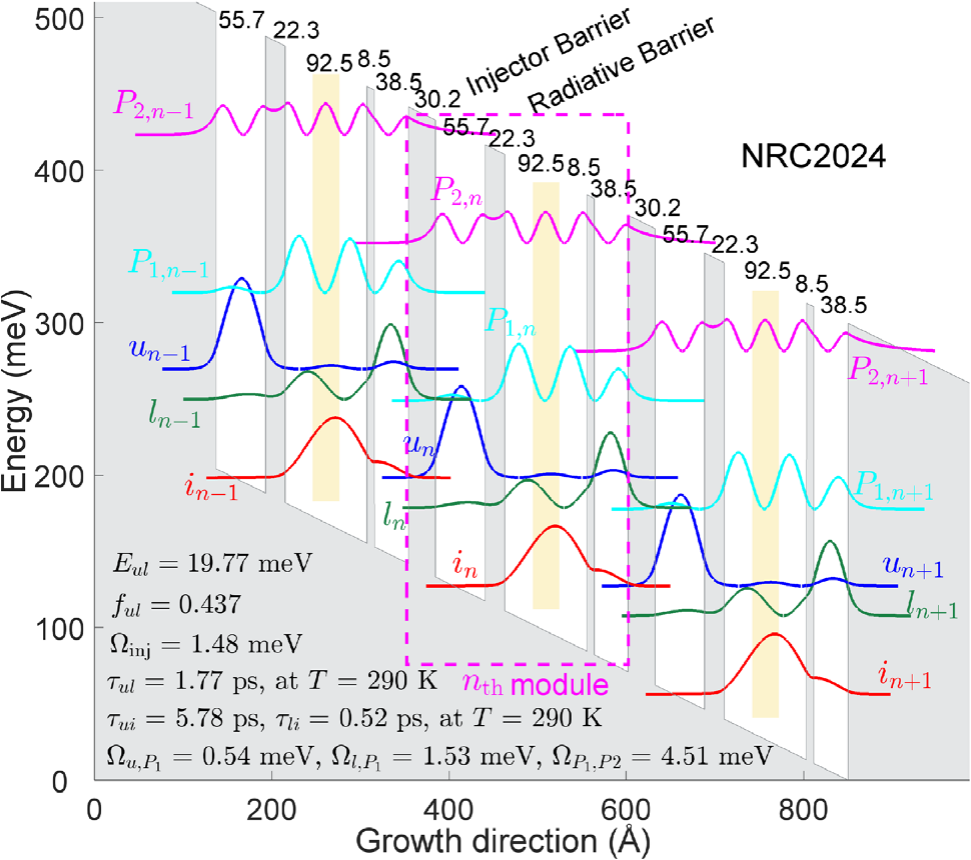
Conduction band diagram and moduli-squared wavefunctions of three neighboring modules at the alignment electric field for a structure based on the three-well direct-phonon scheme. The thicknesses of the quantum wells and barriers, in Angstr$\mathrm {\ddot{o}}$m, starting with the injector barrier, are **30.2**/55.7/**22.3**/92.5/**8.5**/38.5, where the barrier thicknesses are indicated in bold. The yellow section represents the doped area, with a volume doping concentration of 1.5 $\times$ $\mathrm {10^{17}\ cm^{-3}}$ in the central 3 nm region of the widest well.

### Wavefunction engineering of three-well DP structure

In this section, the steps toward the design and optimization of three-well DP structures using double-well phonon are presented. The first step is the selection of the barrier height (conduction band offset) which affects the leakage rate of the electron to the continuum band and also the interface roughness (IFR) scattering time. The successful implementation of high-performance QCLs using 30% and 35% Aluminum fraction in GaAs/AlGaAs material system indicates that either of them are good candidates. However, no obvious improvement is observed in the maximum operating temperature of structure G938 (35% Aluminum) compared to G813 (30% Aluminum) suggesting that designs based on $\mathrm {GaAs/Al_{0.3}Ga_{0.7}As}$ material system have enough barrier height to suppress the electron escaping to the continuum band. Moreover, adding one quantum well and barrier to each module increases the number of interfaces (faster interface roughness scattering), and it may increase the gain broadening of three-well DP structures, proposing that 35% Aluminum may not be a good candidate for this design scheme.

The design process of the three-well DP structure starts with the selection of a double-well phonon in which the thicknesses of two quantum wells and one barrier can be freely changed with no limitation until they satisfy the minimum requirement of a desired phonon transition. In this study, the energy spacing of 47-53 meV and the phonon scattering time of less than 0.6 ps at 250 K have been chosen as minimum criteria for a phonon transition. The quantum well thickness is varied from 30 Å to 110 Å in increments of 1 Å, while the quantum barrier thickness is adjusted from 5 Å to 20 Å with the step of 0.2 Å. Even though a fixed electric field of 25 kV/cm is used for the selection of the double-well phonon design, it will be automatically modified in quantum transport modeling to calculate the current density and the gain of the structure. A slight modification in the design electric field (less than 10%) will not dramatically change the phonon transition energy or its scattering time. For each double-well phonon selected, the radiative well, radiative barrier, injection barrier and the electric field are independently varied to find structures with a desired lasing transition (17-19 meV), oscillator strength (0.35-0.45), and the injection coupling (1.2 - 1.6 meV). The selection criteria for the lasing transition (17-19 meV), oscillator strength (0.35-0.45), and injection coupling (1.2-1.6 meV) were chosen to target a historically established low-loss waveguide region around 4 THz, ensure a diagonal optical transition for high-performance structures, and maintain coherent electron transport for efficient carrier injection, respectively. All three-well structures that satisfied the aforementioned minimum requirements of a QCL module will transfer to our in-house transport model to calculate the gain spectrum and current density at the alignment electric field. The position and the value of two-dimensional doping density are fixed in our general search for optimized three-well QCL design. The electron-LO-phonon and interface roughness scatterings are included in our transport model used for a general search of the three-well structure. However, the ion-impurity scattering is added for the final gain and current density calculation. The electron temperature in our RE-DM model is assumed to be 80 K higher than the lattice temperature for all subbands included in the calculation. While this simplification may not impact the final selection of the quantum design, it significantly reduces the calculation time and enables the general search to be conducted within a reasonable time frame.

Our search engine tool analyzed $\sim$300000 structures in a 5-day timescale and saved important parameters, including the maximum current density, the maximum gain at different temperatures, the coupling and detuning energy between states, the oscillator strength, the scattering time between some states, and the alignment electric field for each structure. The gain of the structure is not the only measure for the selection of the best structures because RE-DM may select a very narrow structure with high injection coupling which suffers from leakage between non-neighboring modules not included in our model. Moreover, no limitation is defined in the selection of the narrowest barrier (the only limitation is the minimum value of 5 Å) which results in the selection of many high performance structures with a very narrow barrier. Previous successful experiences in implementing scattering-assisted THz QCLs based on the $\mathrm {GaAs/Al_{0.25}Ga_{0.75}As}$ material system involved very narrow barriers (6 Å in V843^31^, 5 Å in V962^54^, and 3.5 Å in V895^55^) grown using the molecular beam epitaxy (MBE) machine at the National Research Council (NRC). Despite this demonstrated capability, we decided not to select any structures with barriers thinner than three monolayers in our initial attempt.

### NRC2024 design

Among all structures with high enough gain at 250 K, one of them is selected and presented in this study for further analysis. Figure 2 shows the band diagram and moduli-squared wavefunctions of three modules at alignment electric field in which the detuning energy between the injection state and the upper lasing state is minimized ($\Delta _{i_{n-1},u_n} \approx 0$). There are a few differences between the wavefunctions of the three-well structure in Fig. 2 and those shown in Fig. 1. The second parasitic state of module *n*+1 ($P_{2,n+1}$) is not aligned with the first parasitic state of module *n* ($P_{1,n}$) and they are separated by an approximate detuning energy of 32 meV ($\Delta _{P_{1,n},P_{2,n+1}}$ = -32 meV in NRC2024 vs. $\Delta _{P_{1,n},P_{2,n+1}}$ = 1.5 meV and 0.16 meV in G652 and G813, respectively). The high detuning energy between the two states suggests a very low rate of electron injection even at high temperatures (second-order tunneling approach^26^). The co-alignment of $i_{n-1}\ \rightarrow u_n$ and $l_n \rightarrow P_{1,n+1}$ is another feature of this structure that may enhance carrier depopulation from the lower lasing state, consistent with observations reported in certain structures in Ref.^56^. Even though we did not define any specific figure of merit to be optimized in our general quantum design search (No in-house or commercial optimization tools, e.g Genetic Algorithm, were used to find this structure), a design with double depopulation was among structures with high gain at 250 K.

**Figure 3:**
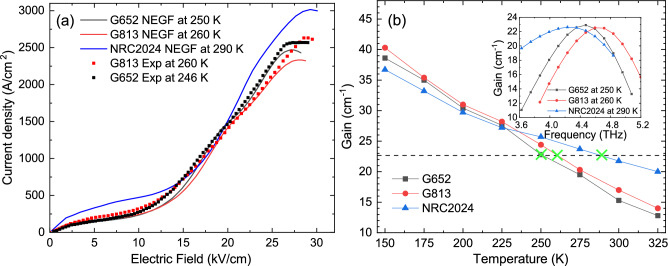
(**a**) The current density of the lasers vs. electric field at a temperature close to the maximum operating temperature. Solid lines are the simulation results using NEGF and the dotted lines are the experimental data extracted from Ref.^17^. (**b**) Maximum gain of the structures vs. temperature, obtained using NextNano.NEGF. The green cross symbols indicate the estimated maximum operating temperature, with the dashed line representing the threshold gain. The inset shows the gain spectrum of the structures at the estimated maximum operating temperature.

To further analyze the proposed three-well DP structure and compare its performance with state-of-the-art two-well structures (G652 and G813), the NEGF simulation tool from NextNano is employed to calculate the gain spectrum and the current density of the structure at different temperatures and electric fields. For a fair comparison, the simulation parameters employed in this study were kept consistent with those used for structures G652 and G813^16,17^. The conduction band discontinuity (CBD), which defines the depth of the quantum well, is assumed to be 300 meV for all structures based on $\mathrm {GaAs/Al_{0.3}Ga_{0.7}As}$. The mean height of the roughness ($\Delta$) and the correlation length ($\Lambda$) that can affect the interface roughness scattering and, consequently, the gain spectrum are 0.08 nm and 8 nm, respectively. The volume doping concentration of $\mathrm {1.5 \times 10^{17} cm^{-3}}$ in 3 nm region at the centre of widest well is used for all structures which is equivalent to the total two-dimensional doping density of $\mathrm {4.5 \times 10^{10}\ cm^{-2}}$ (The simulation parameters used in NEGF and our RE-DM model are the same.). It has been shown that a sheet doping density of $\mathrm {4.5 \times 10^{10}\ cm^{-2}}$ is optimal for maximizing gain in THz QCL structures based on a direct population scheme^57^.

It has been demonstrated that NextNano.NEGF can predict the current density of two-well DP structures at high temperatures by feeding the CBD and IFR parameters proposed in Ref.^17^. A similar approach is used in this study to predict the population of each state and the current density of the selected three-well structure and compare them with the experimental data and the simulation results obtained in G652 and G813. Figure 3a illustrates the current density of three structures (G652, G813, and NRC2024) calculated by the NEGF module at the estimated maximum operating temperature. For comparison, the experimental data (extracted from References^16,17^) are also plotted in dotted lines. The maximum current density of structure NRC2024 is higher than that of structures G652 and G813. The coupling between state $i_{n-1}$ and $u_n$ in NRC2024 is 1.48 meV which is slightly higher than the injection coupling in G652 (1.16 meV) and G813 (1.32 meV). A simple comparison indicates that the increase in coupling injection from NRC2024 to G813 ($\Omega _{iu}^\textrm{NRC2024}/\Omega _{iu}^\textrm{G813}$ = 1.12) is almost the same as the increase from G813 to G652 ($\Omega _{iu}^\textrm{G813}/\Omega _{iu}^\textrm{G652}$ = 1.14) which does not justify the increase in the current density of NRC2024. The effect of IFR scattering on the current density of NRC2024 at low temperatures, particularly its impact on the laser’s threshold current, is more pronounced compared to two-well designs due to the presence of two additional interfaces in each module. However, the increase in the maximum current density of NRC2024 at a temperature close to the maximum operating temperature is less attributed to IFR. The tunneling time between states in two neighboring modules and the LO phonon scattering time inside one module make the main contribution to $J_\textrm{max}$ at a temperature close to $T_\textrm{max}$. Contrary to G652 and G813, in which the injection from $i_{n-1}$ to $u_n$ is the only coherent path between two neighboring module, NRC2024 benefits from the extra leakage ($l_n$ to $P_{1,n+1}$) which depopulates the lower lasing state. The high coupling between the two aligned states at the design electric field ($\Omega _{l_n,P_{1,n+1}}$ = 1.53 meV) is the main player for the high current density. A detailed discussion on the contributions of different tunneling paths to the total current density of the lasers presented in this study can be found in the supplementary material.

**Figure 4:**
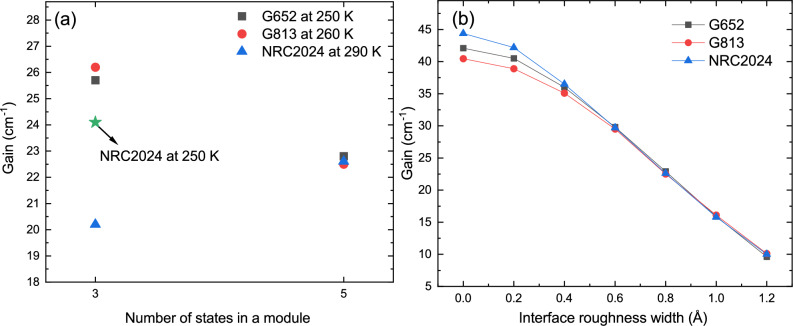
(**a**) Maximum gain of the structures vs. the number of states in each module, calculated using NextNano.NEGF. (**b**) Maximum gain of the structures vs. the mean height of roughness, also calculated using NextNano.NEGF. All calculations were performed at their respective maximum temperatures—experimental (250 K for G652 and 260 K for G813) or estimated (290 K for NRC2024).

### Gain analysis of direct phonon structures

The gain spectrum of the THz QCL is a critical parameter—if not the most important—that requires precise calculation for informed decisions regarding the selection of the structure for MBE growth and fabrication. Although NEGF is well-regarded for its ability to accurately simulate experimental devices due to its comprehensive inclusion of most scattering mechanisms, it may fail if the input parameters—such as IFR parameters and CBD—deviate significantly from the actual values in the fabricated structures. The gain spectrum of G652, G813, and NRC2024 at different temperatures was calculated using NextNano.NEGF and its maximum are plotted in Fig. 3b. The inset shows the gain spectrum of the structures at maximum experimental (250 K for G652 and 260 K for G813) or estimated (290 K for NRC2024) operating temperatures. In the NEGF simulation, the gain value of $\mathrm {\sim 22.5\ cm^{-1}}$ is used as the threshold gain to estimate the maximum operating temperature of the THz QCLs. The gain spectrum of the laser at $T_\mathrm {{max}}$ indicates that the lasing frequency—defined as the frequency at which the gain spectrum is maximized—is nearly identical across all structures, resulting in comparable cavity losses for the three structures. It is visually apparent that the gain spectrum of NRC2024 is broader than those of G652 and G813, likely due to two factors. First, the alignment of levels $l_n$ and $P_{1,n+1}$ with a high coupling energy ($\Omega _{l_n,P_{1,n+1}}$ = 1.53 meV) broadens the gain spectrum. A similar phenomenon has been observed in three-well resonant phonon structures, where the lower lasing state is depopulated via a resonant tunneling process^58^. Second, the intrasubband IFR scattering time in lasing states is shorter in NRC2024 compared to G652 and G813 because of the inclusion of a narrow quantum barrier within the wide phonon well. A comparison of the maximum gain between the structures shows that G813 consistently exhibits a slightly higher gain than G652, with both reaching a gain value of approximately 22.5 cm$\phantom{0}^{-1}$ at their maximum operating temperature. In contrast, NRC2024 does not achieve the highest gain at low temperatures, but its gain decreases at a slower rate, resulting in a higher gain beyond 225 K, eventually reaching 22.6 cm$\phantom{0}^{-1}$ at 290 K.

Further analysis of the gain spectrum behavior will provide insights into the key factors affecting the performance of the structures. This paper proposes that two-well DP structures are limited by the alignment of levels $$P_1$$ and $$P_2$$ with the upper and lower lasing states, and suggests using wavefunction engineering with a three-well design to improve laser performance. The impact of parasitic states can be examined by controlling the number of states included in the NextNano.NEGF simulation. The gain for each structure was calculated at a current near $$J_\mathrm {{max}}$$ and at the maximum operating temperature, as illustrated in Fig. 4a. G813 and G652 exhibit the highest gain when only the first three states are considered in the simulation, achieving gains nearly 25% higher than that of NRC2024 (25.7 cm$$\phantom{0}^{-1}$$ for G652 and 26.2 cm$$\phantom{0}^{-1}$$ for G813, compared to 20.1 cm$$\phantom{0}^{-1}$$ for NRC2024). The gain for NRC2024 was also recalculated at 250 K and is indicated by a green star symbol in Fig. 4a. The data clearly show that G652 and G813 outperform NRC2024 under conditions where the structures behave as clean three-level systems. Including levels $$P_1$$ and $$P_2$$ reduces the gain for G652 and G813, whereas the opposite effect is observed for NRC2024. Specifically, pulling level $$P_1$$ down to align with the LLS enhances extraction efficiency and increases the depopulation rate. Conversely, pushing $$P_2$$ to a higher state decreases the extraction rate from the ULS. It is noteworthy that although six bound states are present in structures G652 and G813, NextNano.NEGF simulations indicate that the sixth state has a negligible effect on the results, as it is populated by only $$\sim$$0.5% of the total electron density. Gain spectrum analysis for NRC2024 shows that the positive effect of parasitic levels is negligible at low temperatures, as indicated by a gain value of 36.7 cm$$\phantom{0}^{-1}$$ at 150 K when only three states are included in the NEGF simulation (compared to 36.9 cm$$\phantom{0}^{-1}$$ when the first five states are included at 150 K, as shown in Fig. 3b). The positive impact of parasitic levels in NRC2024 structure becomes more pronounced at temperatures above 225 K, where the improvement in gain is approximately 0.7 cm$$\phantom{0}^{-1}$$ (26.5 cm$$\phantom{0}^{-1}$$ with three states vs. 27.2 cm$$\phantom{0}^{-1}$$ with five states at 225 K). The difference between the gain values for NRC2024 with three and five states included in the NEGF model increases with temperature, reaching 2.4 cm$$\phantom{0}^{-1}$$ at 290 K (20.2 cm$$\phantom{0}^{-1}$$ with three states vs. 22.6 cm$$$$\phantom{0}^{-1}$$ with five states). The general wavefunction engineering approach employed in this study identified a structure in which parasitic states not only did not degrade the laser’s performance but also enhanced the gain.

### Effect of interface roughness

The comparison between the structures requires careful consideration, as the number of quantum wells and barriers differs between NRC2024 and G813. To analyze the effect of the extra barrier due to interface roughness scattering on the performance of NRC2024 in comparison with G813 and G652, the gain of each structure is calculated at the maximum operating temperatures for a variety of interface parameters changing from 0 Å to 1.2 Å and its maximum is plotted in Fig. 4b. The uncertainty in the mean interface height used in our simulation arises from two possible factors. First, the quality of the interface is influenced by the MBE machine, the growth recipe, the precision of wafer temperature control, the temperatures of the Al, Ga, and As cells, and various other parameters that may vary from one growth to another within the same MBE machine^59^. Second, the IFR parameters estimated for two-well DP designs are fitted from threshold current measurements at low temperatures, and these estimates are only valid for two-well structures grown on that specific MBE machine^17^. The gain values for structures G813, G652, and NRC2024 started at 40.5 cm$$$$\phantom{0}^{-1}$$, 42.1 cm$$$$\phantom{0}^{-1}$$, and 44.4 cm$$$$\phantom{0}^{-1}$$, respectively, and all converged to approximately $$\sim$$22.5 cm$$$$\phantom{0}^{-1}$$ when the mean roughness height reached 0.8 Å. As expected, the impact of interface roughness on NRC2024’s performance is more pronounced than in G652 and G813 when $$\mathrm {\Delta }$$ is less than 0.6 Å. All structures show a comparable performance in terms of the gain value for the change in $$\Delta$$ from 0.6 Å to 1.2 Å meaning that the modification in the maximum operating temperature due to the quality of the MBE growth (small deviation in $$\Delta$$ ) will be the same for G652, G813, and NRC2024. Even though the absolute value of $$\Delta$$ is not determined for an MBE machine, a huge change in $$\Delta$$ from growth to growth using the same MBE machine is very rare^17,60^. Evidence of this assumption is a statement in Ref.^17^ mentioning that the regrowth of design G813 results in a similar behaviour. It is important to note that IFR engineering was not a primary objective in our search for a high-performance three-well DP structure using our in-house RE-DM model. Nevertheless, we decided to minimize the risk of failure by selecting a structure with reliable output (No barrier less than 3 mono layers).

**Figure 5:**
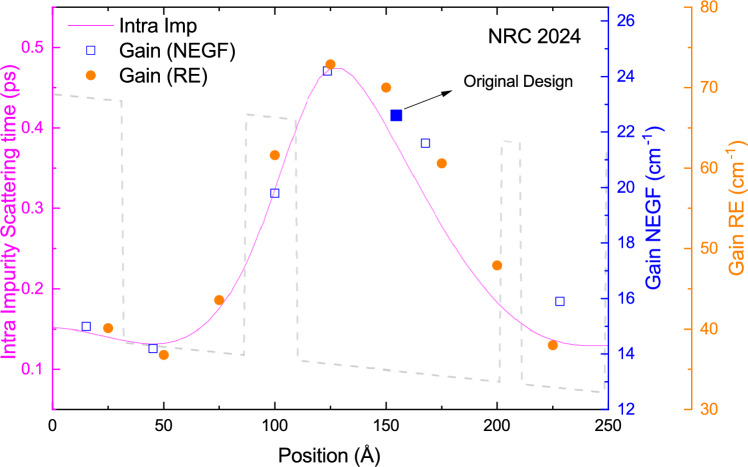
Intrasubband impurity scattering time between the lasing states (solid pink line), peak gain spectrum at the estimated maximum temperature using NextNano.NEGF (blue squares), and in-house RE-DM model (orange circles) versus the position of the dopant. In all calculations in this study, a 3 nm region is homogeneously doped with a volume doping of 1.5 $\times$ $\mathrm {10^{17}cm^{-3}}$ resulting in two-dimensional doping concentration of 4.5 $\times$ $\mathrm {10^{10}\ cm^{-2}}$.

### Doping position engineering

Another critical factor that can impact the structure’s performance, and was not included in our general search, is the dopant positioning within the quantum structure. Several studies have shown that performance improvements can be achieved through careful engineering of the doped region^17,61–64^. However, many of these studies are hindered by a lack of precise information on doping levels and distribution, largely due to doping segregation effects. In this section, we propose a simplified yet effective method for identifying the optimal dopant position in NRC2024 structure. This method can be integrated into our search algorithm to simultaneously optimize both the quantum structure and the dopant position with minimal additional complexity in the MATLAB code implementation. It is well-documented that impurity scattering due to dopants in THz QCLs can significantly impact the gain spectrum, particularly the gain bandwidth^20,65^. Although a full quantum transport model is the most accurate approach to assess the effect of dopant positioning on gain broadening, the intrasubband impurity scattering time between the lasing states can serve as a useful decision-making parameter. The intrasubband scattering time between the lasing state for ion-impurity scattering (pink solid line), the maximum value of the gain using our in-house RE-DM model (orange circles), and NextNano.NEGF (blue squares) are calculated and the results are illustrated in Fig. 5. Our calculations indicate that the minimum gain and impurity scattering times occur when the dopant is positioned in the radiative well, while the maximum corresponds to the dopant being located in the widest well immediately following the radiative barrier. The maximum gain of the structure increases from $\sim$14 $\mathrm {cm^{-1}}$ ($\sim$38 $\mathrm {cm^{-1}}$) to $\sim$24 $\mathrm {cm^{-1}}$ ($\sim$72 $\mathrm {cm^{-1}}$) using NEGF in NextNano (in-house RE-DM model) when the position of the doping moves from the radiative well to the phonon well. Modifying the dopant position or its distribution within a structure does not significantly affect the wavefunction or energy spacing between states (except for the effect of the Hartree potential), suggesting that the intrasubband IFR scattering time between the lasing states remains nearly constant and does not contribute to additional broadening of the gain spectrum or dephasing time between the aligned states. Additionally, the influence of dopant positioning on state population near the maximum operating temperature is minimal, as the inter-subband ion-impurity scattering time is much longer than the inter-subband LO-phonon scattering time at a temperature above 250 K. The minimal change in the population of each state due to the change in doping position is based on an assumption that the change is dephasing time between the aligned states (for example levels $i_{n-1}$ and $u_{n}$) would not affect the coherent tunneling regime (This is a valid assumption for NRC2024 with a coupling injection of $\Omega _{i_{n-1},u_n} = 1.5$ meV). These considerations suggest that using the intrasubband ion-impurity scattering time between lasing states as a criterion for optimizing dopant position is reasonable. Figure 5 shows that approximately 10% improvement in gain value can be achieved when the dopant position of the original design (NRC2024) moves toward the radiative barrier. The same behavior was observed in Ref.^17^ for structures labeled G938 LD and CD. It is important to mention that all the presented results in this study, including the IV and the gain calculation (Figs. 3 and 4), are based on the original structure with the doping position at the centre of the widest well. The relatively good agreement between the behavior of the intrasubband ion-impurity scattering time and the calculated gain indicates that the dopant position can also be incorporated into our general search approach with minimal complexity in the calculations.

**Figure 6:**
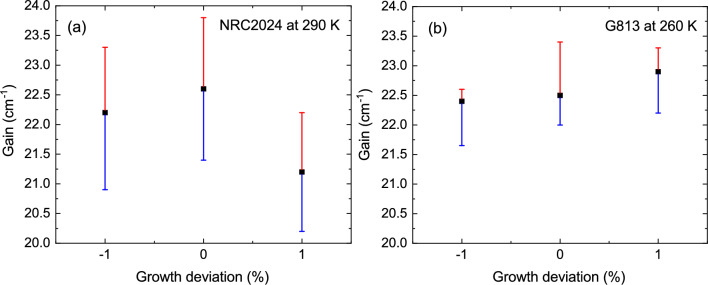
(**a**) Maximum gain of NRC2024 vs. growth deviation, calculated using NextNano.NEGF at the estimated maximum operating temperature (*T* = 290 K). (**b**) Maximum gain of G813 versus growth deviation, calculated using NextNano.NEGF at the maximum operating temperature (*T* = 260 K). The red and blue bars represent the change in gain value when the doping concentration is adjusted to 5 $\times$ $\mathrm {10^{10}\ cm^{-2}}$ and 4 $\times$ $\mathrm {10^{10}\ cm^{-2}}$, respectively.

### Effect of MBE drift

One of the benefits of the DP structure compared to the resonant-phonon structure, as discussed in the introduction, is that each module only requires one alignment, which simplifies the design process. However, our proposed three-well DP structure, selected through wavefunction engineering, requires two alignments $i_{n-1} \rightarrow u_n$ and $l_n \rightarrow P_{1,n+1}$ to occur simultaneously at the same electric field. This raises concerns about the stability of the laser and its sensitivity to MBE growth deviations (i.e., variations in quantum well/barrier thickness and doping concentration). Any deviations in layer thickness or doping level alter the quantum states and may disrupt the simultaneous injection and extraction alignments, thereby impairing laser performance. In this section, the gain of structures NRC2024 and G813 is calculated and presented in Fig. 6, assuming that the quantum structure and doping concentration vary by $\pm 1\%$ and $\pm 10\%$, respectively. The black squares represent the gain values for the original quantum design (position 0), as well as for structures with 1% thinner modules (position -1) and 1% thicker modules (position +1). The NEGF simulation indicates that any deviation in the quantum structure thickness results in a reduction in gain for NRC2024. However, G813 exhibits a slightly increased gain for wider modules. The gain change for G813 is less than 0.5 $\mathrm {cm^{-1}}$, whereas for NRC2024, it exceeds 1 $\mathrm {cm^{-1}}$. It is important to note that a 1% change in quantum well and barrier thickness represents the worst-case scenario and is unlikely to occur during the MBE growth of GaAs-based THz QCLs. For example, in G813, the module thickness deviation was 0.15%^17^, which is nearly an order of magnitude smaller than the assumed deviation in this calculation. The gain variation in NRC2024 would be less than 0.2 $\mathrm {cm^{-1}}$ if a 0.15% MBE drift is assumed. The red and blue bars depict the change in gain when the doping concentration is adjusted to $5 \times 10^{10}\ \mathrm {cm^{-2}}$ and $4 \times 10^{10}\ \mathrm {cm^{-2}}$ ($\pm 10\%$ deviation from the nominal value of $4.5 \times 10^{10}\ \mathrm {cm^{-2}}$), respectively. The effect of doping concentration on the performance of both G813 and NRC2024 is similar, with both structures exhibiting higher gain when the doping concentration is $5 \times 10^{10}\ \mathrm {cm^{-2}}$. The $\pm 10\%$ variation in doping concentration is based on the accuracy of measurement techniques, such as C-V measurement, and may not necessarily reflect real growth variations. Our previous experience of observing consistent I-V characteristics of fabricated lasers from wafers grown in two separate growth campaigns using the same MBE machine confirms that the doping concentration change is much less than 10%.

## Conclusion

This study introduces a fast and reliable quantum transport model that enables QCL designers to explore various structures and evaluate their performance in terms of maximum gain across different temperatures. Through a detailed analysis of two high-temperature THz QCLs (G652 and G813) based on a two-well direct phonon scheme, a three-well structure was developed using a generalized wavefunction engineering method and rate equation-density matrix transport model, with its performance assessed using NextNano.NEGF. The observed improvement in gain compared to G652 and G813—primarily attributed to the enhanced alignment between the lower lasing state and the parasitic state—suggests that achieving room-temperature THz QCL is within reach. Furthermore, the investigation into the impact of interface roughness scattering parameters and dopant positioning on the performance of the structure demonstrates that the gain can be further enhanced through a careful optimization process. The close agreement between NextNano.NEGF and our in-house RE-DM formalism for NRC2024 structure confirms the robustness of our comprehensive quantum design search, ensuring that no high-performance structure is overlooked. The QCL research community is encouraged to undertake the experimental implementation and validation of the proposed NRC2024 structure to assess its practical performance and potential applicability.

## Methods

In this study, two different transport models—the in-house rate equation-density matrix (RE-DM) model and the commercial software NextNano.NEGF—were employed to analyze the performance of the structures based on the direct phonon (DP) depopulation scheme. Both models effectively predicted the laser performance in terms of current density and maximum operating temperature. In our RE-DM model, the energy states and wavefunctions of all structures were calculated using the Transfer Matrix Model (TMM) due to its ease of implementation, computational efficiency, and ability to incorporate band nonparabolicity without recursive calculations. Nonparabolicity was included by assuming an energy-dependent effective mass for the electron, updating it for each energy state based on the model presented in Ref.^66^. This approach allowed for calculating the corresponding wavefunctions with minimal computational overhead. The oscillator strength calculation in this work is based on the model presented in Ref.^47^, where an energy-dependent effective mass was used to incorporate nonparabolicity into a single-band treatment using equation 8 in Ref.^47^. It should be noted, however, that the conduction band wavefunctions included in this calculation are normalized using equation 14 from Ref.^47^. These normalized wavefunctions were subsequently used for other calculations, including coupling strength, and both elastic (interface roughness and ion-impurity) and inelastic (optical phonon) scattering times. All NEGF simulation results in this study were generated using the NEGF module in NextNano software. Identical simulation parameters, including the conduction band discontinuity (CBD = 300 meV) and the interface roughness settings ($\Delta$ = 0.08 nm and $\Lambda$ = 8 nm), are used in both RE-DM and NextNano simulation models. The scattering mechanisms included in NEGF simulations are optical phonon, interface roughness, ion-impurity, and electron-electron scatterings.

## Supplementary Information

Supplementary Information.

## Data Availability

The datasets used and/or analyzed in this current study are available from the corresponding author on reasonable request.
