# Supplementary material for: Wavefunction engineering towards high-performance terahertz quantum cascade lasers
Source: Sci Rep. 2025 Aug 11;15:29349. doi: 10.1038/s41598-025-10080-4 (PMC12340077; doi:10.1038/s41598-025-10080-4)
Supplement: Supplementary file 1 — Supplementary Information. [file 41598_2025_10080_MOESM1_ESM.pdf]

Supplementary Materials
Wavefunction Engineering towards High-performance Terahertz Quantum Cascade Lasers

Seyed Ghasem Razavipour[1,*]

[1]Quantum and Nanotechnologies Research Center, National Research Council Canada, 1200 Montreal Road, Ottawa, Ontario K1A 0R6, Canada
[*]Ghasem.Razavipour@nrc-cnrc.gc.ca

Introduction

Two quantum transport models—the rate equation-density matrix (RE-DM) model and the non-equilibrium Green's function (NEGF) model—were used in this manuscript to design and analyze the performance of terahertz (THz) quantum cascade lasers (QCLs) based on the direct phonon (DP) depopulation scheme. The detailed methods for parameter calculation in the RE-DM model including coupling strength, leakage to parasitic states (P_1 and P_2), leakage from upper lasing state to injector state, the current density are presented and compared with the simulation results obtained using NextNano.NEGF.

Coupling strength calculation

The scattering times among states within a QCL module and the tunneling times between modules must be accurately calculated to predict the current density of the laser using our RE-DM model. Calculating the coupling between states (half of the anticrossing gap) and the pure dephasing time is crucial, as these parameters define the coherence of the tunneling process. In this study, the method presented in Ref. [1] is employed to determine the coupling, with results for several recently published structures shown in Table S1. To evaluate the accuracy of these calculations, the coupling strength reported in Ref. [2] and the coupling energy from Ref. [3], extracted from the Hamiltonian transferred to the EZ basis, are also included in Table S1. All structures, except ETH2019, are based on the $GaAs/Al_{0.3}Ga_{0.7}As$ material system, assuming a conduction band discontinuity (CBD) of 300 meV. The data presented in Table S1 indicate that the method used to calculate the coupling strength is accurate and that the current density predicted by the RE-DM model is reliable.

	ETH2019	G552	G652	Lund2022	G813	NRC2024
Ref. [3]	1.58	1.08	1.17	1.49	N/A	N/A
Ref. [2]	N/A	1.07	1.15	N/A	N/A	N/A
This study	1.54	1.06	1.16	1.48	1.32	1.48

Table. S1. The coupling between the injector state (i) and the upper lasing state (u) at alignment electric field for structures based on DP scheme. The anticrossing energy presented in Table 1 of the Ref. [2] is equivalent of 2Ω in this study and data reported in Ref[3]

.

As suggested in Ref. [3], increasing coupling injection may enhance the performance of DP structures. However, the impact of coupling injection on gain broadening should also be considered, particularly when utilizing a narrow injection barrier. Additionally, a very narrow injector layer may increase the tunneling rate between non-adjacent modules due to wavefunction expansion.

Parasitic states P1 and P2 in two-well DP structures

One of the primary motivations for exploring an alternative design based on the DP scheme was the limitation of two-well structures in engineering parasitic states P_1 and P_2. As explained in the main text, the positions of states P_1 and P_2 are

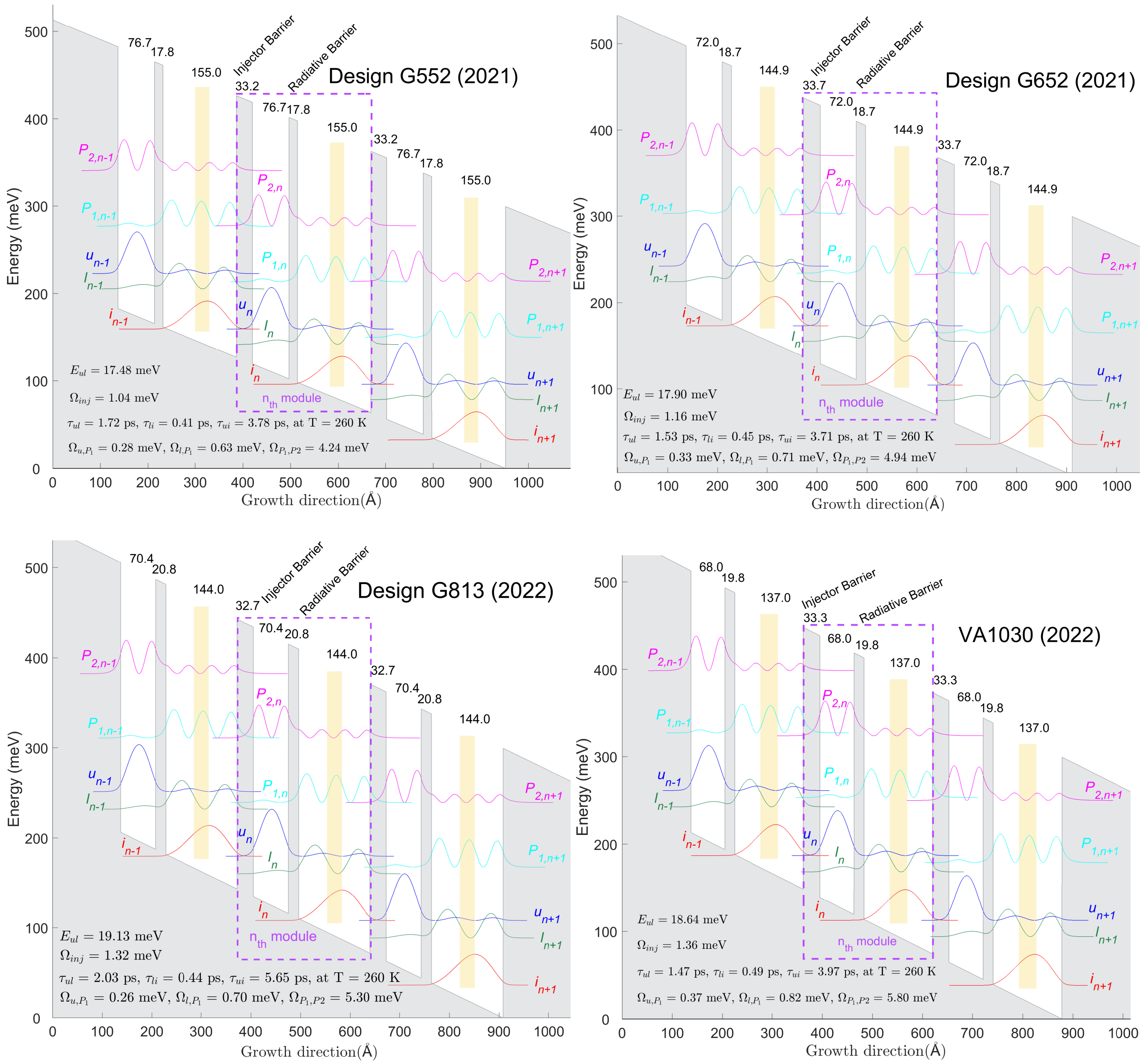

Figure. S1. Conduction band diagram and the moduli-squared wavefunctions of three neighboring modules at alignment electric fields for THz QCL active regions based on direct phonon scheme. The thickness of each quantum well and barrier in Angström is shown on top of the graph. The central 3 nm of the largest well is n-doped with 4.5×10^{10} cm^{-2} (yellow section).

determined by the thicknesses of the phonon well and the radiative well, respectively. Three high-performance structures (G552, G652, and VA1030), each with different phonon well thicknesses and phonon energy spacings ranging from 46 meV to 55.7 meV, are presented in Table 1 of the main text. A more thorough comparison between the presented designs can be made by illustrating the wavefunctions of these structures. Figure S1 shows the conduction band diagrams and the moduli-squared wavefunctions of three neighboring modules in G552, G652, G813, and VA1030.

Despite significant differences in the quantum structures of G552 and VA1030 compared to G652 and G813 , no notable variation was observed in the relative positioning of states $P_{1,n}$ and $P_{2,n+1}$ with respect to states u_{n-1} and l_{n-1}. A high coupling strength between $P_{1,n}$ and $P_{2,n+1}$, which are nearly aligned at the designed electric field, forms a mini-band with an anticrossing gap (2Ω) exceeding 10 meV . These highly coupled states partially align with the upper and lower lasing states of the previous module, thereby acting as a leakage source. The coupling between $P_{1,n}$ and $P_{2,n+1}$ increases from 4.24 meV to 5.8 meV as the injection coupling ($\Omega_{i,u}$) increases from 1.04 meV to 1.36 meV in structures G652 and VA1030 , respectively. As detailed in Ref. [4], selecting the figure of merit $\zeta = \frac{\Omega_{i_{n-1},u_n}}{\Omega_{u_n,P_{1,n+1}}}$ favors designs with a narrower injector barrier and a thicker radiative barrier, resulting in lasers with lower oscillator strength (transition from G652 to G813). However, reducing the oscillator strength has its limitations, as it broadens the gain spectrum and may result in insufficient gain at high temperatures. To enhance the performance of THz QCLs based on the DP scheme, a new structure with greater flexibility in controlling the position of parasitic states is necessary.

Parasitic states P1 and P2 in three-well DP structures

In this section, examples of structures based on three-well DP designs are presented, and their key features will be investigated. The band diagram and moduli-squared wavefunctions of the structures in Table 2 of the main text are illustrated in Figure. S2. The energy spacing in the phonon wells (E_{li}) and the lasing energy (E_{ul}) for all structures are $\sim$50 meV and $\sim$18 meV, respectively. However, the relative position of state $P_{1,n+1}$ with respect to state u_n can be engineered such that the detuning energy ($\Delta_{u_n,P_{1,n+1}}$) changes from $\sim$-23 meV in Example 1 to +28 meV in Example 4. The location of the narrow barrier in the phonon well and its thickness define the shape of the wavefunctions and also the position of the third state in double-well phonon (state $P_{1,n}$). Placing the barrier close to the centre of phonon well (Example1) will push the third state ($P_{1,n+1}$) to a higher energy such that it will not couple with either upper (u_n) or lower (l_n) lasing state of the previous module. The same strategy was employed in structures VB0837[5] ,VB0843 and VB0847[6] to minimize the leakage current from upper and lower lasing states to the parasitic states thereby achieving a "clean" three-level system. Our general search approach examined all possible double-well phonon configurations with the aim of identifying a structure with high performance at temperatures above 250 K. These four structures are purposely selected to demonstrate that minimizing the leakage to states P_1 and P_2 should not be the sole criterion for QCL design. A simplistic analysis may suggest that Example1 is the best structure due to a fairly clean 3-level system, However, the NextNano.NEGF simulations show that the best two structures are Example 2 and 4. The gain of these structures are calculated at 250 K using NextNano.NEGF and illustrated in Figure S3. The data indicate that Example 3 exhibits inferior performance compared to the other structures, primarily due to the alignment of states u_n and $P_{1,n+1}$ at the design electric field. An experimental implementation of DP scheme that suffers from an alignment between the upper lasing state (u_n) and the first parasitic state ($P_{1,n+1}$) is VB0747[7]. However, the coupling between the two state is very low ($\Omega_{u_n,P_{1,n+1}}$ = 0.07 meV) because of employing a very low coupling injection and oscillator strength. It may not be straight-forward or fair to compare the performance of VB0747 with other two-well DP structures based on $GaAs/Al_{0.3}Ga_{0.7}As$ material system because they exhibit fundamentally different features. For instance, the injection coupling and the oscillator strength of VB0747 (Ω_{i_{n-1},u_n} = 0.9 meV, f_{ul} = 0.08, extracted from Table 5.3 in Ref. [8]) are significantly lower than those of G652 (Ω_{i_{n-1},u_n} = 1.16 meV, f_{ul} = 0.3, extracted from Table 1 in Ref. [2]) primarily due to the use of a very thick injector (38 Å in VB0747 vs 33.7 Å in G652) and radiative (31 Å in VB0747 vs 18.7 Å in G652) barriers. Moreover, the lasing frequencies of the two structures are significantly different resulting in a different cavity losses (2.4 THz in VB0747 vs. 4.1 THz in G652).

Leakage path from upper lasing state to injector state

Direct phonon depopulation scheme had not been considered a potential candidate for high performance THz QCL until the clean 3-level system was proposed in 2017[9]. One main concern was the wrong depopulation of upper lasing state due to a wavefunction overlap between levels u and i, particularly in vertical structures with high oscillator strength. This leakage path can be controlled by employing a low oscillator strength and a large phonon energy gap, a strategy used in all high-performance QCLs based on the direct phonon structure. Regulation of this leakage pathway is crucial for enhancing the maximum operating temperature, a topic discussed in detail in the main body of the paper. A detailed comparison between this leakage pathway and the operating temperatures of structures reported in recent literature would offer valuable insights, underscoring the necessity of careful monitoring and controlling of this parameter. Table S2 demonstrates the percentage of the electron relaxing from

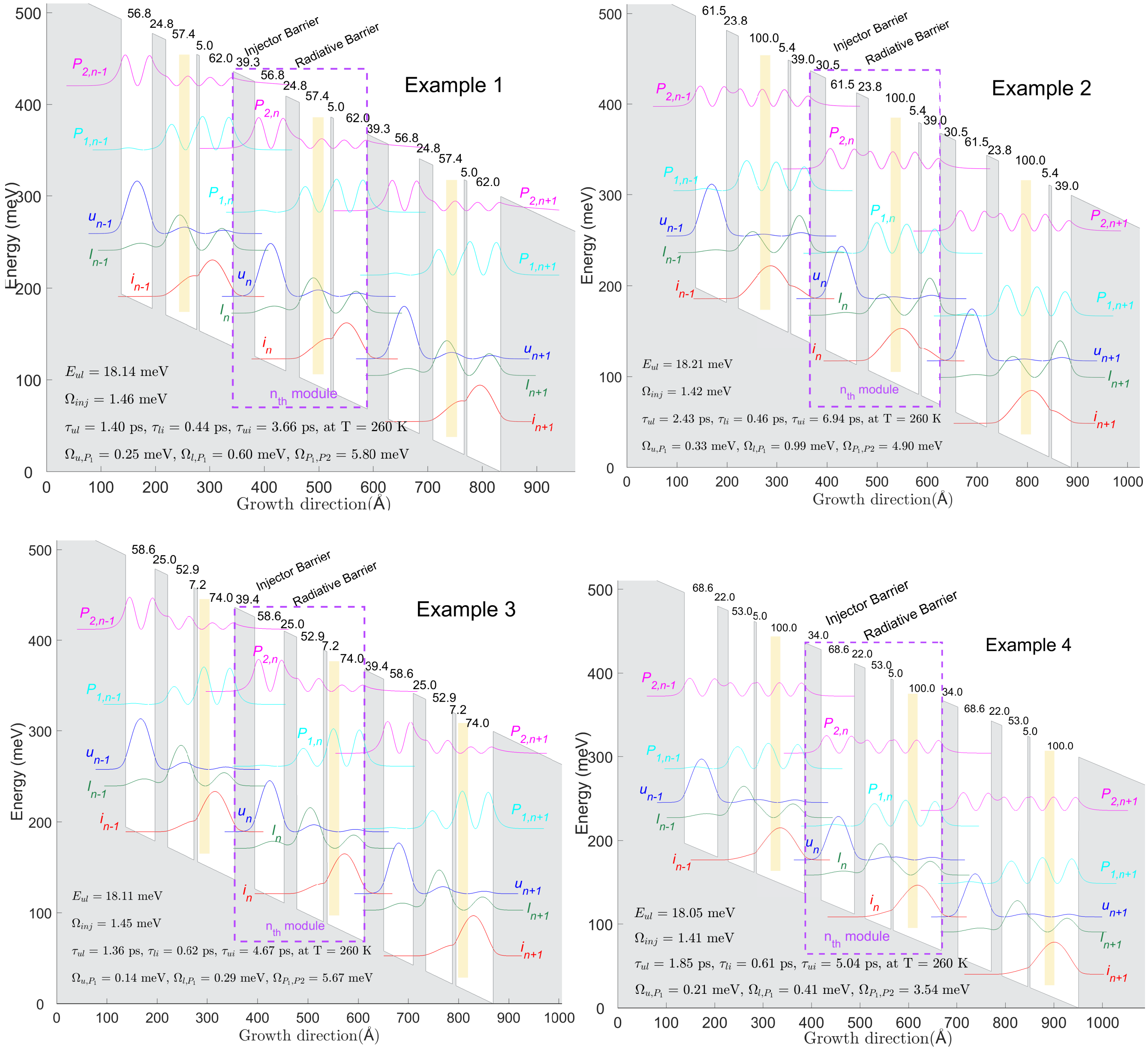

Figure. S2. Conduction band diagram and the moduli-squared wavefunctions of three neighboring modules at alignment electric fields for THz QCL active regions based on direct phonon scheme. The thickness of each quantum well and barrier in Angström is shown on top of the graph. The yellowy section indicates the doped area, with a volume doping of 1.5×10^{17} cm^{-3} in the 3 nm region of the double-well phonon.

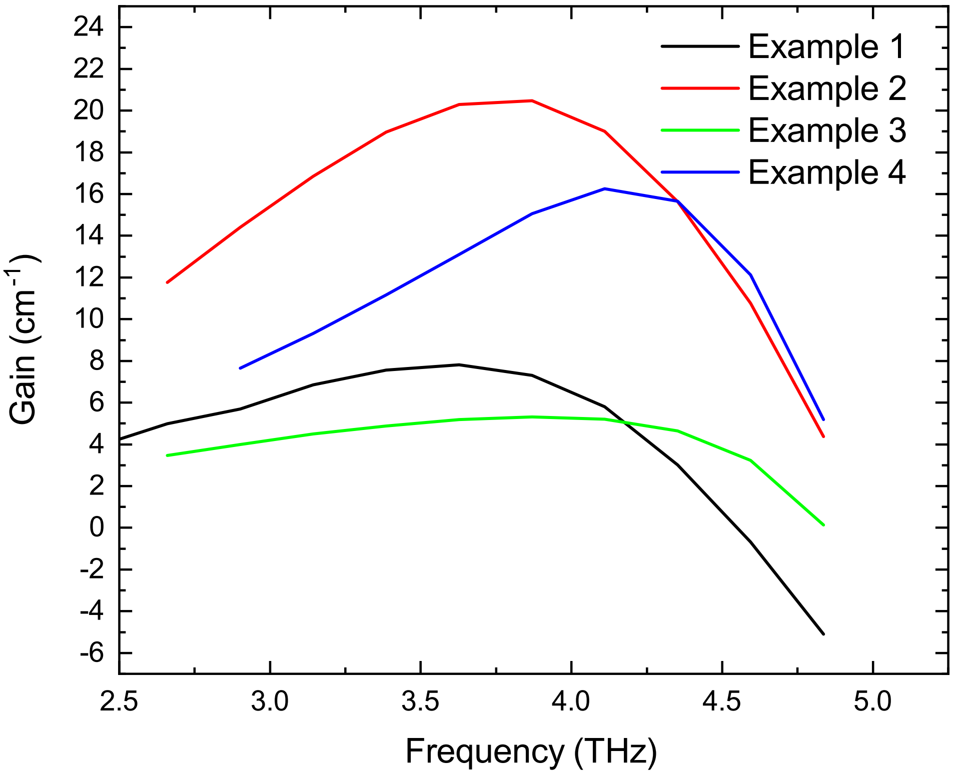

Figure. S3. The gain spectrum of the structures illustrated in Figure S2 using NextNano.NEGF at 250 K.

upper lasing state to the injector state at a temperature close to the maximum operating temperature of the laser, along with the maximum operating temperatures for comparison. Although this leakage path is not the sole factor influencing laser performance, Table S2 shows that a lower leakage rate correlates with a higher operating temperature. NRC2024 has not yet been experimentally implemented; therefore, no experimental data for its maximum operating temperature are available. However, calculations using NextNano.NEGF indicate that NRC2024 is capable of generating sufficient gain to operate at approximately 290 K. Consequently, the leakage rate for NRC2024 was also evaluated at 290 K.

	ETH2019	G552	G652	G813	NRC2024
Leakage	33.96%	29.6%	26.9%	24.27%	21.8%
T_{max}	210 K (exp.)	225 K (exp.)	250 K (exp.)	260 K (exp.)	290 K (sim.)

Table. S2. The percentage of the electron relaxed from upper lasing state to the injector state at a temperature close to the maximum operating temperature of the lasers. The second row shows the maximum operating temperature of the lasers. NRC2024 is not yet experimentally implemented.

.

Current density of the lasers

The current density-voltage (J-V) characteristics of lasers at various temperatures, particularly at low temperatures, provide crucial information that helps QCL designers better understand quantum transport in the structure. For instance, a comprehensive study was conducted on various THz QCL samples using NEGF modeling in which the experimental maximum current density was compared with the simulation data using the same set of parameters[10]. This study suggested that samples from different laboratories cannot be compared using a same set of parameters in the simulation. Later, they showed that different conduction band offsets (CBOs) can help in evaluating the performance of two-well DP structure and proposed a new design with a high gain at 265 K[3]. Another example is the estimation of interface roughness parameters for two-well DP devices based on the $GaAs/Al_{0.3}Ga_{0.7}As$ material system grown in Waterloo and fabricated at MIT[4]. They demonstrated that using the threshold current density of the laser at very low temperature and comparing it with the simulation data from NEGF will give us a set of

interface roughness parameters that would predict the performance of the laser. However, there are limitations how to use those parameters for different design schemes.

The current density-voltage simulation curves for G813, G652 and NRC2024 using NextNano.NEGF are illustrated in Figure.3 of the main manuscript. The calculated maximum current density for NRC2024 at a temperature close to the maximum operating temperature is higher than that of G652 and G813. Considering that the effect of injection coupling between the state i and u on current density is not dominant for devices with coherent injection ($4\Omega_{iu}^2\tau_u\tau_{\|iu}$ = 3.48 meV at 290 K for NRC2024), the source of such high current requires further exploration. Table S3 presents the maximum current density for these structures calculated using NextNano.NEGF and our RE-DM model. The interface roughness parameters used in both simulations are identical; however, e-e scattering time is not included in our RE-DM model. It has been reported that excluding e-e scattering leads to an underestimation of current density and an overestimation of gain (It has a more detrimental impact on material gain than on current density), which can be adjusted by modifying the interface roughness (IFR) parameters[11]. This strategy of IFR parameter modification was not employed in this study (a higher Δ was used in Refs. [12–14] to predict the IV of 3P-QCLs). The current densities of the lasers calculated using RE-DM modeling is always lower compared to those estimated using NEGF by a maximum of ∼3% indicating that RE-DM modeling is sufficiently accurate for predicting the performance of the lasers.

	$J_{max,G652}(A/cm^2)$	$J_{max,G813}(A/cm^2)$	$J_{max,NRC2024}(A/cm^2)$
NextNano.NEGF	2472	2333	3015
RE-DM	2446	2256	2945

Table. S3. The maximum current density of the lasers at a temperature close to the maximum operating temperature using NextNano.NEGF and RE-DM model

.

In our transport model, the first five states in each module are included, and the current densities of all possible tunneling paths are calculated. The various contributors to the total current density, extracted from our RE-DM modeling, are summarized in Table S4. Although the maximum current density of NRC2024 is higher than that of G652 and G813, this additional current does not originate from the primary tunneling path ($i_{n-1} \rightarrow u_n$). Notably, the current from the lower lasing state to the first parasitic state ($J_{l_{n-1},P_{1,n}}$ = 774 A/cm^2) accounts for approximately 26% of the total current, explaining the high population inversion in NRC2024 and, consequently, its higher gain. Another key observation is current leakage from states u_n and l_n to $P_{1,n+1}$ in G652 and G813. Device G813 demonstrates improved electron extraction efficiency due to a lower coupling between states u_n and $P_{1,n+1}$ (0.26 meV in G813 vs. 0.33 meV in G652). In both lasers, approximately 100 A/cm^2 is injected from the upper and lower lasing states to the first parasitic state (60 + 40 A/cm^2 in G652 vs. 21 + 76 A/cm^2 in G813). However, the higher ratio of $J_{l_{n-1},P_{1,n}}/(J_{u_{n-1},P_{1,n}} + J_{l_{n-1},P_{1,n}})$ in G813 (78%) compared to G652 (60%) indicates that $P_{1,n}$ depopulates more efficiently from the lower lasing state in G813.

	J_{i_{n-1},u_n}	J_{i_{n-1},l_n}	$J_{u_{n-1},P_{1,n}}$	$J_{l_{n-1},P_{1,n}}$	$J_{P_{1,n},P_{2,n+1}}$	J^{RE}_{total}
G652	2170	19	40	60	128	2446
G813	1996	12	21	76	126.5	2256
NRC2024	1978	14	31	774	33	2945

Table. S4. Main contributors to the total current density of the lasers, calculated using the RE-DM model at temperatures close to the maximum measured (G652 and G813) and estimated (NRC2024) operating temperatures.

.

References

1. Bastard, G. *Wave mechanics applied to semiconductor heterostructures* (New York, NY (USA); John Wiley and Sons Inc., 1990).
2. Khalatpour, A., Paulsen, A. K., Deimert, C., Wasilewski, Z. R. & Hu, Q. High-power portable terahertz laser systems. *Nat. Photonics* **15**, 16–20 (2021).
3. Rindert, V., Önder, E. & Wacker, A. Analysis of high-performing terahertz quantum cascade lasers. *Phys. Rev. Appl.* **18**, L041001 (2022).
4. Khalatpour, A. *et al.* Enhanced operating temperature in terahertz quantum cascade lasers based on direct phonon depopulation. *Appl. Phys. Lett.* **122** (2023).
5. Albo, A., Flores, Y. V., Hu, Q. & Reno, J. L. Split-well direct-phonon terahertz quantum cascade lasers. *Appl. Phys. Lett.* **114**, 191102 (2019).
6. Lander Gower, N., Piperno, S. & Albo, A. The significance of carrier leakage for stable lasing in split-well direct phonon terahertz quantum cascade lasers. *Photonics* **7** (2020).
7. Lander Gower, N., Levy, S., Piperno, S., Addamane, S. J. & Albo, A. Exploring the effects of molecular beam epitaxy growth characteristics on the temperature performance of state-of-the-art terahertz quantum cascade lasers. *Sci. Reports* **14**, 17411 (2024).
8. Khalatpour, A. *New frontiers in THz quantum cascade lasers*. Ph.D. thesis, Massachusetts Institute of Technology (2020).
9. Albo, A., Flores, Y. V., Hu, Q. & Reno, J. L. Two-well terahertz quantum cascade lasers with suppressed carrier leakage. *Appl. Phys. Lett.* **111**, 111107 (2017).
10. Winge, D. O., Franckie, M. & Wacker, A. Simulating terahertz quantum cascade lasers: Trends from samples from different labs. *J. Appl. Phys.* **120** (2016).
11. Demic, A. *Density matrix modelling of terahertz frequency quantum cascade lasers: Steady state analysis and maxwell-bloch dynamics*. Ph.D. thesis, University of Leeds (2019).
12. Dupont, E. *et al.* A phonon scattering assisted injection and extraction based terahertz quantum cascade laser. *J. Appl. Phys.* **111** (2012).
13. Razavipour, S. G. *et al.* An indirectly pumped terahertz quantum cascade laser with low injection coupling strength operating above 150 k. *J. Appl. Phys.* **113** (2013).
14. Razavipour, S. G. *et al.* A high carrier injection terahertz quantum cascade laser based on indirectly pumped scheme. *Appl. Phys. Lett.* **104**, 041111 (2014).
